# Diagnostic accuracy of MRI–CBCT fused images in assessment of clinically diagnosed internal derangement of the temporomandibular joint

**DOI:** 10.1007/s11282-023-00727-1

**Published:** 2024-01-17

**Authors:** Ethar M. ElShennawy, Walaa M. Hamed, Sahar M. Samir

**Affiliations:** Oral and Maxillofacial Radiology Department, Faculty of Dentistry, Ain Shams University, Cairo, Egypt

**Keywords:** Joint disease, Temporomandibular, Joint disc, Cone beam computed tomography, MRI scan

## Abstract

**Objectives:**

To evaluate the diagnostic accuracy of Fused (MRI)–CBCT images in the assessment of internal derangement of the temporomandibular joint.

**Methods:**

MRI and CBCT images of the TMJ were evaluated bilaterally in 10 patients with clinically diagnosed internal derangement. Image fusion was performed using Amira 3D Software (version 5.4.3, Thermo Fisher Scientific Inc.).

**Results:**

The AUC index for MRI–CBCT fused images was 0.83, which was significantly different from the null hypothesis value of 0.5. This was confirmed by inter-examiner reliability index of 0.87, which is statistically significant.

**Conclusion:**

MRI–CBCT fused images can significantly improve the accuracy and inter-examiner reliability in the evaluation of clinically diagnosed cases with internal derangement.

## Introduction

Temporomandibular joint (TMJ) disorders (TMDs) are a prevalent health issue affecting millions of people worldwide and usually involve pain and dysfunction in the joint. Among the various types of temporomandibular disorders, TMJ internal derangement is the most common, accounting for 41.1% of TMD patients [1]. Diagnostic imaging plays a crucial role in evaluating patients with TMD. It is used to confirm suspected conditions, rule out diseases, and provide more information when the clinical diagnosis is ambiguous. There are certain historical and clinical factors that can increase the chances of detecting abnormalities through imaging. For instance, the presence of a reciprocal click, closed lock, or crepitus may be indicative of underlying issues [2].

According to the Wilkes Stages Classification of Internal Derangement (Stages I–V), which classifies internal derangement according to the clinical presentation, radiographic findings and anatomical co-relation, the last two stages encircle the incidence of bony changes that occurs beyond disc displacement, hence the importance of investigation of osseous changes and not only the disc-condyle relationship [3]. Therefore, in internal derangement cases, it is essential to assess the overall shape of the condyle, glenoid fossa and articular eminence and to confirm that the cortical borders are uniform and intact. Hard tissue changes on the images may be depicted as flattening, sclerosis, osteophyte formation, erosion, and presence/absence of loose bodies within the joint space [4].

MRI is the reference method for imaging the soft tissue components of the temporomandibular joint complex and is the best imaging modality in diagnosing disc displacements [5]. MRI also detects the early signs of TMJ dysfunction, such as thickening of the anterior or posterior band, rupture of retro-discal tissue, changes in the shape of the disc, joint effusion and, to a lesser extent, osseous changes [6]. The standard scanning sequences were T1-weighted, T2-weighted, and proton-density (PD) images were obtained. The PD images serve to visualize the disc-condyle relationship, while T2-weighted images typically diagnose joint inflammation in the joint [7, 8].

Currently, CBCT is becoming popular due to its potential advantages of high spatial resolution in comparison to 2D panoramic radiography and lower radiation dose and cost than medical computed tomography. Moreover, they provide multiplanar reconstructed images of the region of interest and have overcome the limitations of magnification, distortion, and superimposition of anatomical structures that usually occur in panoramic images [9, 10]. The ability to assess details in multiplanar views makes CBCT a unique tool for accurate and precise evaluation of dentoalveolar structures. The high spatial resolution of CBCT allows for the evaluation of early bony changes in the TMJ, such as osteophyte formation, sub- condylar sclerosis, thinning and erosion of the condylar head [11, 12]. However, due to different limitations, neither CBCT nor MRI alone provides enough information for the internal derangement of the TMJ, which includes both hard and adjacent soft tissues. In certain clinical cases, it is valuable to be able to visualize both soft and hard tissues simultaneously [13].

Introduced in the early 1990s, the multimodal image fusion technique involves combining medical images from different sources to create a more reliable and precise image for clinical diagnosis and treatment. This technique has many applications in clinical practice and can help clinicians make better-informed decisions, ultimately leading to better patient outcomes [14]. CT/CBCT-MRI fusion has been widely used in the maxillofacial region, including diagnosing cranial and spinal diseases, chronic otitis media, nasopharyngeal carcinoma and other jaw tumors, in addition to diagnosis and treatment planning for orthognathic conditions [15–18].

The fusion of CBCT and MRI images, which aids in diagnosing bone-related issues and soft tissue abnormalities, offers a valuable chance to enhance the examiner's understanding of joint anatomy. In the interpretation of TMJ using PD-weighted and T1-weighted MRI, commonly employed techniques, the articular disc exhibits a low signal intensity similar to that of the cortical bone surrounding the condylar head, glenoid fossa, and articular eminence. In certain instances, when the joint space is reduced, the articular disc comes into close proximity with the cortical boundaries of the condylar head or the posterior slope of the articular eminence. These tissues share a similar low signal and can be easily mistaken, leading to errors or inconsistencies in the diagnosis of internal derangements. Furthermore, in severe cases of TMJ degeneration, alterations in the size and shape of the articular disc, as well as increased bone marrow signal, can hinder the identification of joint structures. By registering MRI and CBCT images, the opportunity arises to mitigate these common sources of diagnostic errors in TMJ-MRI interpretation. Al-Saleh et al. proved that the CBCT-MRI fused image not only increases the intra- and interxaminer reliability in the assessment of internal derangement of TMJ but also increases the accuracy of novice examiners’ assessment of disc positions, as it allows for simultaneous evaluation of both the soft tissue and bony components of the TMJ complex [19, 20].

Hence, the primary objective of our present study is to evaluate the precision of utilizing combined MRI–CBCT images for diagnosing internal derangement-related findings such as disc displacement, joint effusion, and associated bone alterations.

## Materials and methods

### Patients

A power analysis was designed to have adequate power to apply a 2-sided statistical test of the research hypothesis (null hypothesis) that the accuracy and reliability of the intrinsic MRI–CBCT co-registered images for the diagnosis of temporomandibular joint effusion and osseous changes in patients with anterior disc displacement is below 90%. According to the results of Al-Saleh, Mohammed AQ, et al. 1, in which the inter-examiner reliability was 97%, and by adopting an alpha (*α*) level of 0.05 (5%) and a beta (*β*) level of 0.20 (20%), i.e., power = 80%, the predicted sample size (*n*) was a total of 20 joints, i.e., 10 patients. The sample size was not the sole choice of the author.

Ten adult patients (20 TMJs) with a history of TMJ pain and noises on jaw movement or function, which were confirmed upon clinical examination by a TMD specialist at the Oral and Maxillofacial Surgery Clinic, were recruited for investigation. The Human Research Ethics Committee (855) approved the study. Informed consent was obtained from all study participants. All patients had MRI and CBCT images obtained at the same visit with the teeth in maximum intercuspation using polyvinylsiloxane occlusal bite stents.

*Inclusion criteria* Male/female patients with an age range between 25 and 50 years old diagnosed with TMJ internal derangement with at least two of the following symptoms:

- Crepitus on mouth opening.
- Clicking sound on mouth opening.
- Limited mouth opening.
- Tenderness over the temporomandibular joint on palpation.
- Localized joint pain.
- Popping on joint movement.
- Change in the patient's perception of their bite.
- Deviation of mandible with mouth opening
- Locked jaw with wide mouth opening
- Swelling of the face on the affected side.

*Exclusion criteria*:

- History of trauma.
- History of TMJ surgery.
- Vulnerable groups.
- Pregnant females.
- Patients with pacemakers or metal vascular clips.
- Patients who are unable to remain motionless in the supine position.

### Image acquisition

#### CBCT protocol

**Fig. 1:**
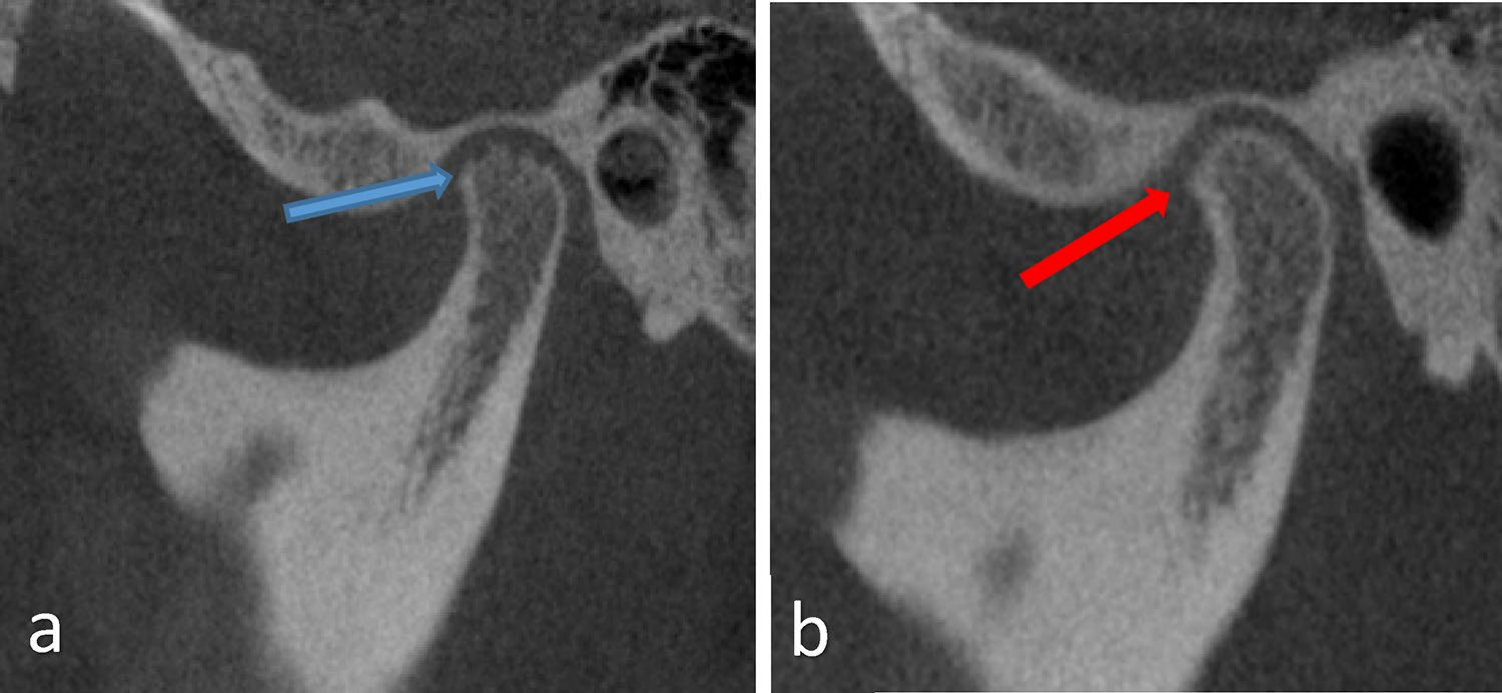
Corrected sagittal CBCT of the right (**a**) and left sides (**b**), blue arrow-showing erosion in the superior border of the condylar cortex, red arrow-showing osteophyte formation and reduced joint space

Each CBCT scan was acquired in 360 degrees of rotation with proper subject upright positioning with the Frankfort plane parallel to the floor and collimated to avoid radiosensitive structures (thyroid and orbits). Scans were performed using an i-CAT scanner (Imaging Sciences International, Hatfield, USA) at a medium field of view setting, 16 cm wide,13 cm height, scan time 26 s and 0.25 mm voxel size. This included the maxilla, mandible, and both TMJ condyles. CBCT images (Fig. 1) allowed for evaluation of the osseous structures: the condylar head and its cortical lining, the glenoid fossa and the articular eminence.

#### MRI protocol

**Fig. 2:**
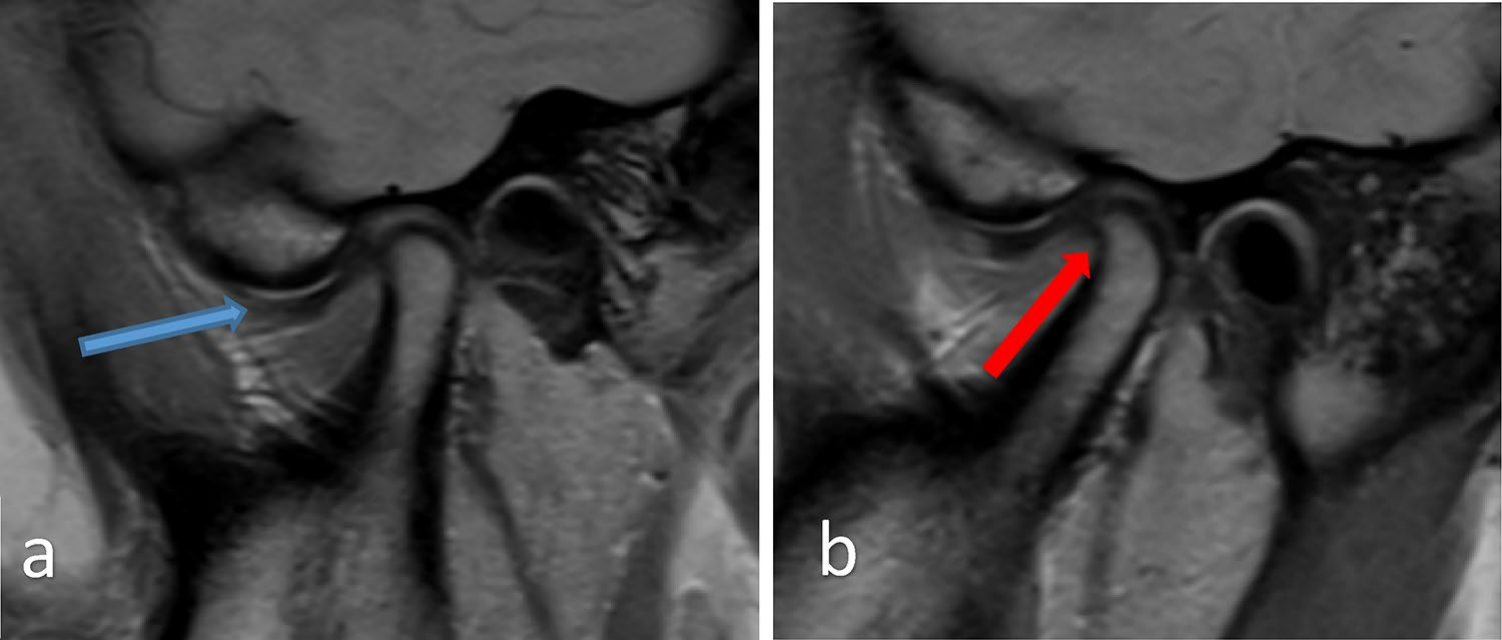
Corrected sagittal proton density-weighted images of the right (**a**) and left sides (**b**) in the closed mouth position. The blue arrow indicates anterior disc displacement and effusion in the superior joint compartment. Red arrow indicating anterior disc displacement, joint effusion in the superior joint compartment, and osteophyte formation is apparent on the anterior surface of the condyle

MRI of the TMJ was performed in the supine position without sedation or intravenous contrast administration by a 1.5 Tesla scanner (Magnetom Sola, Siemens Healthineers, Munich, Germany) with a multichannel head array coil. Small field of view (13 cm × 13 cm), dedicated bilateral closed-mouth and open-mouth oblique sagittal sections were parallel to the long axis of the condyle. In the following sequences, T1 images provide detailed anatomic information of the TMJ, including the articular disc and bony structures. T2-weighted images are sensitive to water content and can help visualize the soft tissues of the TMJ, including the articular disc, synovial fluid, and surrounding muscles. T2-weighted images can help identify disc displacement, inflammation, and other soft tissue abnormalities. Finally, proton density-weighted images (Fig. 2) provide a balance between T1- and T2-weighted images and can help identify both bony and soft tissue abnormalities of the TMJ.

### Imaging fusion

**Fig. 3:**
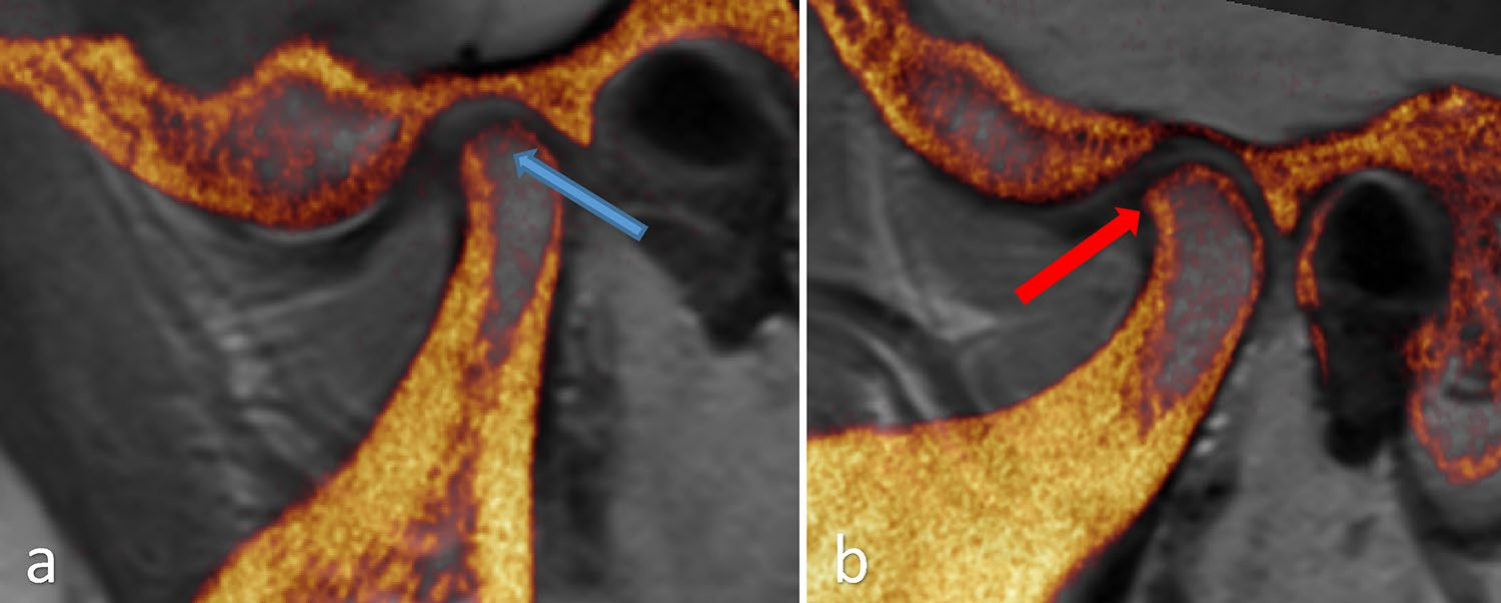
Right (**a**) and left (**b**) oblique sagittal view of the MRI–CBCT fused image. The PD MRI is the primary image, and the CBCT is the overlay image demarcated with an orange/yellow gradient. The blue arrow shows the CBCT overlay with thinning of the condylar cortex, and the primary PD-MRI shows anterior disc displacement and joint effusion in the superior joint compartment. Red arrow—showing osteophyte formation on the anterior surface of the condyle demarcated by the CBCT overlay, and the primary PD-MRI shows anterior disc displacement

Image fusion of CBCT and MRI was carried out using Amira visual software (version 5.4.3, ThermoFisher Scientific Inc.), and the registered process was carried out in the sub-application multiplanar viewer module. Because of the differences in the patient positioning and different sizes of the acquisition matrix in both image sets, a rigid transform model and the similarity metric of normalization mutual information were used for registration in the present study so that the CBCT images would co-align with MRI images. The CBCT-MRI fused images were demonstrated in a color-coded manner. The overlapped structures from MRI appeared in grayscale, and structures from CBCT appeared in bright orange color. The final registered image will allow for simultaneous evaluation of both MRI and CBCT volumes. (Fig. 3).

### Image evaluation and scoring

Two radiologists with at least 5 years of experience in oral and maxillofacial radiology evaluated each of the data sets (MRI, CBCT and MRI–CBCBT images) independently, meaning that each radiologist evaluated 60 images for a total of 20 TMJs. Each radiologist graded the MRI images as follows: (0) for normal disc position and (1) for anterior disc displacement in the closed mouth position. Joint effusion was graded (0) if absent and (1) for visible joint effusion.

The CBCT images were graded (0) if no evident osteoarthritic changes were visible and (1) if any of the following bony changes were present (osteophyte formation, thinning of the condylar cortex, sclerosis of the condylar cortex, joint mice and/or sub-condylar Ely’s cysts formation).

The fused image (done only in the closed mouth position) is graded as follows: disc displacement is scored if anteriorly displaced in the closed mouth position (1) and (0) for normal disc position. Joint effusion is scored (0) if absent and (1) if present, and any of the previously mentioned osteoarthritic changes is scored (0) if absent and (1) if present.

### Statistical analysis

Categorical data are presented as frequency and percentage values and were analyzed using McNemar’s test. Diagnostic accuracy was determined using receiver operating characteristic curve analysis and by calculating sensitivity and specificity values. Interobserver reliability was analyzed using Cohen’s kappa coefficient. The statistical significance of the area under the curve and Kappa coefficient was determined using the *z* test. The significance level was set at *p* ≤ 0.05 within all tests. Statistical analysis was performed with R statistical analysis software version 4.3.0 for Windows [21].

## Results

Ten patients with 20 TMJs were included in this cross-sectional study with a gender predilection of 1:0 (female: 10, male: 0). Their ages ranged from 17 to 38 years, with a mean age of 21.5 years. We examined three specific observations (displacement of the disc, joint effusion, and osseous changes) as these were frequently observed among the patients included in our study.

MRI scanning of 20 joints of the 10 studied cases showed 18 (90.0%) joints affected with anterior disc displacement and seven (35.0%) joints showing signs of effusion. Out of the 20 investigated TMJs, only two showed a normal disc position, where the posterior band of the articular disc was at the 11’o clock position relative to the condylar head. One of the normally positioned discs was flattened with abnormal morphology.

Eighteen TMJs showed anterior disc displacement in the closed mouth position. In all 18 joints, the disc was completely anterior to the condylar head, 13 showed complete flattening of the displaced disc, three showed crumpled disc morphology, and two showed normal disc morphology. Twelve joints showed reduced joint space. Effusion was recorded in 7 out of the 20 joints; in four joints, effusion was seen in the retro-discal tissues, two joints showed effusion in both superior and inferior joint compartments, and one joint only showed effusion in the superior joint compartment. Table 1 shows the descriptive data for the MRI findings.

**Table 1:** Descriptive data for MRI findings

Parameter	Status		Value
Disc position	Normal position	*n*	2
		%	10.0%
	Anterior disc displacement	*n*	18
		%	90.0%
Joint effusion	No signs of effusion bilaterally	*n*	13
		%	65.0%
	Any sign of effusion	*n*	7
		%	35.0%

CBCT scanning of the 20 joints showed only one joint to be free of osseous changes. Flattening of the condylar head was seen in eight joints, thinning of the condylar cortex was seen in five joints, loss of the integrity of the condylar cortex was recorded in two joints, and osteophyte formation was seen in seven joints. Joint mice were only evident in one joint. Abnormal condylar morphology caused by erosion of the condylar head was seen in five joints, erosion occurred posteriorly in one joint, and pencil thin condyle was observed in the four other joints. Ely’s cyst formation was observed in two joints, and only two joints showed sclerosis in the articular eminence. However, reduced joint space was evident in eight out of the 20 joints. Table 2 shows the descriptive data for the CBCT findings.

**Table 2:** Descriptive data for CBCT findings

Parameter	Status		Value
Osseous changes	Absent	*n*	1
		%	5.0%
	Present	*n*	19
		%	95.0%

The fused images where then investigated for the disc displacement, joint effusion and osseous changes simultaneously. Table 3 shows the descriptive data for the fused image findings.

**Table 3:** Descriptive data for fused images findings

Parameter	Status		Value
Disc position	Normal position	*n*	2
		%	10.0%
	Anterior disc displacement	*n*	18
		%	90.0%
Joint effusion	No signs of effusion	*n*	12
		%	60.0%
	Any sign of effusion	*n*	8
		%	40.0%
Osseous changes	Absent	*n*	2
		%	10.0%
	Present	*n*	18
		%	90.0%

The diagnostic accuracy was measured for disc displacement and joint effusion changes in the fused images in comparison to the MRI images alone (Table 4). Regarding disc position, there was no significant difference between diagnosis made with the two methods (*p* = 1). Sensitivity value was (89.40%) (i.e. indicating the probability of detecting a true positive) and specificity value was (0.0%) (i.e. indicating the probability of detecting a true negative). The Area Under the Curve value was (0.444) (i.e. indicating the probability of true diagnosis) which was non-statistically significantly different from the null hypothesis value of (0.5) (i.e. indicating poor diagnostic ability) (*p* > 0.05). Regarding joint effusion, there was no significant difference between diagnosis made with the two methods (*p* = 1). Sensitivity value was (100.00%) (i.e. indicating the probability of detecting a true positive) and specificity value was (92.31%) (i.e. indicating the probability of detecting a true negative). The area under the curve value was (0.962) (i.e. indicating the probability of true diagnosis) which was statistically significantly different from the null hypothesis value of (0.5) (i.e. indicating good diagnostic ability) (*p* < 0.001).

**Table 4:** Diagnostic accuracy for MRI measurements

Measurements	MRI alone	Fused image	Not diseased	Diseased	*p*-value	Sensitivity (95% CI)	Specificity (95% CI)	AUC (95% CI)
Disc position	Not diseased	n	0	2	1ns	89.4% (82.0%:96.8%)	0.00% (0.00%:0.00%)	0.444 (0.370:0.519)ns
		%	0.0%	10.0%				
	Diseased	n	2	16				
		%	10.0%	80.0%				
Joint effusion	Not diseased	n	12	1	1ns	100.00% (100.00%:100.00%)	92.31% (77.82%:106.79%)	0.962 (0.886:1.000)*
		%	60.0%	5.0%				
	Diseased	n	0	7				
		%	0.0%	35.0%				

*Significant (*p* ≤ 0.05) ns; non-significant (*p* > 0.05)

The diagnostic accuracy was also measured for the osseous changes in the fused images in comparison to the CBCT images only (Table 5). There was no significant difference between diagnosis made based on the index and the actual diagnosis (*p* = 1). Sensitivity value was (89.47%) (i.e. indicating the probability of detecting a true positive) and specificity value was (0.0%) (i.e. indicating the probability of detecting a true negative). The AUC value was (0.447) (i.e. indicating the probability of true diagnosis) which was non-statistically significantly different from the null hypothesis value of (0.5) (i.e. indicating poor diagnostic ability) (*p* > 0.05).

**Table 5:** Diagnostic accuracy for CBCT measurements

Measurements	CBCT alone	Fused images	Not diseased	Diseased	*p*-value	Sensitivity (95% CI)	Specificity (95% CI)	AUC (95% CI)
Osseous changes	Not diseased	*n*	0	1	1ns	89.47% (75.67%:103.27%)	0.00% (0.00%:0.00%)	0.447 (0.000:0.988)
		%	0.0%	5.0%				
	Diseased	*n*	2	17				
		%	10.0%	85.0%				

*Significant (*p* ≤ 0.05) ns; nonsignificant (*p* > 0.05)

**Fig. 4:**
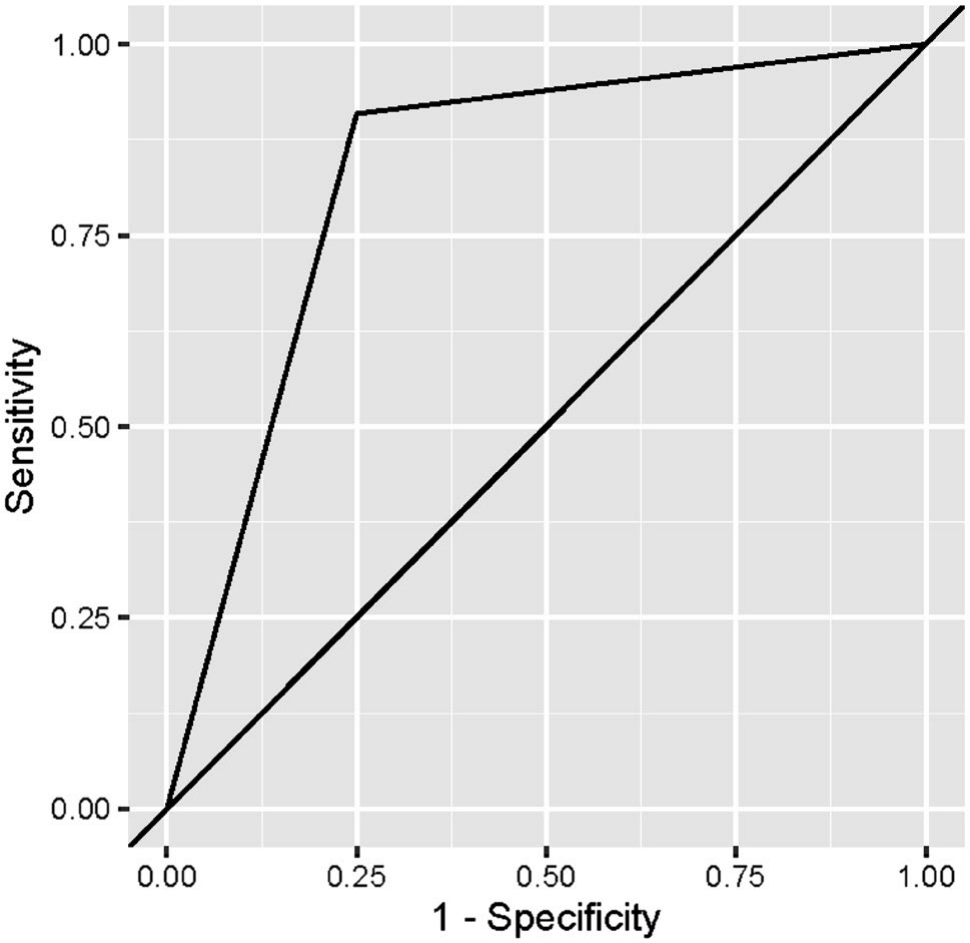
Receiver operator characteristic curve for the overall diagnostic accuracy of the MRI–CBCT image

The overall diagnostic accuracy of the fused images where then calculated against both the CBCT and the MRI images independently. There was no significant difference between the diagnosis made based on the index and the actual diagnosis (*p* = 1). The sensitivity value was 90.91% (indicating the probability of detecting a true positive), and the specificity value was 75.00% (indicating the probability of detecting a true negative). The area under the curve value was 0.830 (Fig. 4) (i.e., indicating the probability of true diagnosis) which was significantly different from the null hypothesis value of 0.5 (i.e. indicating good diagnostic ability) (*p* < 0.001). (Table 6).

**Table 6:** Overall diagnostic accuracy of the fused images

MRI and CBCT findings	Fused images	Not diseased	Diseased	*p* value	Sensitivity (95% CI)	Specificity (95% CI)	AUC (95% CI)
Not diseased	*n*	12	4	1ns	90.91% (82.41%:99.40%)	75.00% (53.78%:96.22%)	0.830 (0.712:0.947)*
	%	60.0%	6.7%				
Diseased	*n*	4	40				
	%	6.7%	66.7%				

*Significant (*p* ≤ 0.05) ns; nonsignificant (*p* > 0.05)

Interobserver reliability is presented in Table 7. There was a strong agreement between measurements made by different observers that was statistically significant (*k* = 0.870, *p* < 0.001).

**Table 7:** Interobserver reliability by the kappa test = 0.87, which is statistically significant

First observer	Second observer	Not diseased	Diseased	Kappa (95% CI)
Not diseased	*n*	14	2	0.870 (0.723:1.016)*
	%	23.3%	3.3%	
Diseased	*n*	1	43	
	%	1.7%	71.7%	

*Significant (*p* ≤ 0.05) ns; nonsignificant (*p* > 0.05)

## Discussion

Although MRI is considered the gold standard for the evaluation of internal derangement of the TMJ, accurate and robust diagnosis is challenging, and it has been reported in the literature that even experienced radiologists show poor reliability when diagnosing TMJ internal derangement on MRI [22]. Anterior disc displacement is assessed by investigating the relationship between the articular disc and the condylar head. Nevertheless, osseous structures may not be clearly visible in MRI. By superimposing MRI images onto CBCT images, a more accurate definition of bone structures can be achieved. This enhances the ability to diagnose the position of the disc relative to the condyles [23]. This is particularly useful in cases of reduced joint space and flattened articular disc, where the low signal disc can be mistaken for the outer cortical lining of the articular eminence or the condylar head [19].

The CBCT–MRI fusion technique is innovative and highly useful in the qualification of patients for diagnosing internal derangement of the TMJ [23]. Generally, mutual information is a similarity measure between floating images and reference images to measure the correlation between them. The greater the mutual information, the higher the correspondence between the images, and the better the fusion effect [13]. This technique overcame the concern of any discrepancy regarding the different condylar positions between the two modalities because of the patient position during the acquisition. When compared to the matching fiducial marker fusion technique, the mutual information theory was proven accurate by all radiologists in a study conducted by Al-Saleh et al*. *[24]

To date, few studies have investigated the accuracy and reliability of fused images in the assessment of the temporomandibular joint. A previous study proved that MRI–CBCT registered images significantly improved the intra- and examiner reliability among experienced readers to evaluate internal derangement of the TMJ compared to MRI alone [19]. Another study also proved the increased diagnostic accuracy of MRI–CBCT images in distinguishing articular disc calcification from the loose body of the temporomandibular joint [13].

In the present study, the estimated overall diagnostic accuracy of the MRI–CBCT fused image represented by the area under the ROC curve (AUC) was significantly higher for the MRI–CBCT image. This indicates that the fused image is favorable in the assessment of internal derangement of the TMJ. This was also endorsed by the excellent interobserver reliability. There was a strong agreement between measurements made by different observers that was statistically significant (*k* = 0.870, *p* < 0.001). The fused image created by outlining and separating osseous structures in the CBCT image from the MRI images is a superior diagnostic tool for internal derangement. It can help determine the position of the disc and detect various osseous changes, even for examiners with limited experience. The findings of this study are consistent with other related studies using the same technique [13, 19, 23].

Fusion of MRI and CBCT allows for comprehensive diagnosis of both bone and soft tissue structures in the same image. In this study, we believe that it is a promising technique that is worthy of investigation for accuracy.

## Conclusion

The multimodal image fusion technique is a feasible and promising technique for evaluating the internal derangement of the temporomandibular joint, allowing for simultaneous evaluation of the soft and osseous components of the joint complex, increasing the diagnostic accuracy and improving inter-examiner reliability.

## Limitations and recommendations

The specific inclusion criteria limited the sample size of the study. A larger sample size is recommended in future studies.
